# Treatment with subcutaneous and transdermal fentanyl: results from a population pharmacokinetic study in cancer patients

**DOI:** 10.1007/s00228-015-2005-x

**Published:** 2016-01-14

**Authors:** Astrid W. Oosten, João A. Abrantes, Siv Jönsson, Peter de Bruijn, Evelien J. M. Kuip, Amílcar Falcão, Carin C. D. van der Rijt, Ron H. J. Mathijssen

**Affiliations:** Department of Medical Oncology, Erasmus MC Cancer Institute, Groene Hilledijk 301, 3075 EA Rotterdam, The Netherlands; Department of Pharmacology, Faculty of Pharmacy, University of Coimbra, Coimbra, Portugal; CNC—Center for Neuroscience and Cell Biology, University of Coimbra, Coimbra, Portugal; Department of Pharmaceutical Biosciences, Uppsala University, Uppsala, Sweden; Netherlands Comprehensive Cancer Organisation, Utrecht, The Netherlands

**Keywords:** Fentanyl, Pharmacokinetics, Subcutaneous, Transdermal, NONMEM

## Abstract

**Purpose:**

Transdermal fentanyl is effective for the treatment of moderate to severe cancer-related pain but is unsuitable for fast titration. In this setting, continuous subcutaneous fentanyl may be used. As data on the pharmacokinetics of continuous subcutaneous fentanyl are lacking, we studied the pharmacokinetics of subcutaneous and transdermal fentanyl. Furthermore, we evaluated rotations from the subcutaneous to the transdermal route.

**Methods:**

Fifty-two patients treated with subcutaneous and/or transdermal fentanyl for moderate to severe cancer-related pain participated. A population pharmacokinetic model was developed and evaluated using non-linear mixed-effects modelling. For rotations from subcutaneous to transdermal fentanyl, a 1:1 dose conversion ratio was used while the subcutaneous infusion was continued for 12 h (with a 50 % tapering after 6 h). A 6-h scheme with 50 % tapering after 3 h was simulated using the final model.

**Results:**

A one-compartment model with first-order elimination and separate first-order absorption processes for each route adequately described the data. The estimated apparent clearance of fentanyl was 49.6 L/h; the absorption rate constant for subcutaneous and transdermal fentanyl was 0.0358 and 0.0135 h^−1^, respectively. Moderate to large inter-individual and inter-occasion variability was found. Around rotation from subcutaneous to transdermal fentanyl, measured and simulated plasma fentanyl concentrations rose and increasing side effects were observed.

**Conclusions:**

We describe the pharmacokinetics of subcutaneous and transdermal fentanyl in one patient cohort and report several findings that are relevant for clinical practice. Further research is warranted to study the optimal scheme for rotations from the subcutaneous to the transdermal route.

**Electronic supplementary material:**

The online version of this article (doi:10.1007/s00228-015-2005-x) contains supplementary material, which is available to authorized users.

## Introduction

For the treatment of moderate to severe cancer-related pain, strong opioids are the treatment of choice [1, 2]. Fentanyl is a synthetic opioid with a high affinity for the μ-opioid receptor and is 75–100 times more potent than morphine [3, 4]. According to international guidelines, fentanyl is not the opioid of first choice [2], but nonetheless, it is widely used for the treatment of cancer-related pain. Fentanyl is recommended in patients with renal failure [2]. Furthermore, because the incidence of constipation is lower in fentanyl compared to morphine [5–7] and it can be administered through a patch, it is a popular drug for the treatment of cancer-related pain. Fentanyl can also be used if an opioid rotation is necessary after failure on another type of opioid. Its low molecular weight and high lipid solubility make it suitable for transdermal delivery [8]. Although the first patches used a reservoir design carrying risks of drug leakage or abuse, currently available patches have a matrix design. They release fentanyl at a proposed rate of 12.5–100 μg/h and the amount delivered is proportional to the surface area of the patch. As a gradient is needed between the patch and the skin, the patch contains more fentanyl than is released. A mean bioavailability of 92 % (57–146 %) has been reported [9]. Reservoir and matrix patches and different types of matrix patches have been shown to have similar pharmacokinetic profiles [10, 11]. The slow decrease in fentanyl concentrations after transdermal patch removal and the delay before achieving the maximum plasma concentrations (both reflecting slow release of fentanyl) make transdermal fentanyl (patches) unsuitable for fast titration in patients with severe pain. In this setting, parenteral titration is therefore preferred. Subcutaneous administration has been proven to be safe and effective [12, 13] and has advantages over the intravenous route as no vascular access is needed, making it easier to change sites and avoiding complications associated with indwelling intravenous catheters. In addition, subcutaneous administration can also be applied safely in an out-of-hospital setting [14].

In our cancer institute, patients with severe pain are preferably titrated with continuous subcutaneous opioids, and in this setting, fentanyl is frequently used. However, little is known about the pharmacokinetics of subcutaneously (sc) administered fentanyl as opposed to the transdermal (td) route. As part of a larger prospective pharmacologic opioid project, we studied the pharmacokinetics of fentanyl in hospitalized cancer patients with moderate to severe cancer-related pain. The purpose was to study the pharmacokinetics of fentanyl administered via the subcutaneous and transdermal routes to cancer patients. A second aim was to evaluate rotations from the subcutaneous to the transdermal route.

## Patients, materials and methods

Between January 2010 and November 2013, patients admitted to the Erasmus MC Cancer Institute (Rotterdam, The Netherlands) and treated with fentanyl for moderate to severe cancer-related nociceptive pain were asked to participate in the study. Fentanyl Sandoz® Matrix patches were used in available doses of 12/25/50/75/100 μg/h and patches could be combined. Patches were applied to the chest wall or upper arm and were replaced every 72 h. The starting dose in opioid-naive patients was 12 μg/h and doses in other patients were based on previous treatment. In case of severe pain, patients were titrated by continuous sc infusion with the possibility of an extra bolus every hour. The dose of the bolus usually parallels the dose given per hour. Doses were titrated based on clinical effects. When pain control was reached and doses were stabilized, patients could be rotated to fentanyl (td) patches depending on the clinical setting. For the rotation of sc to td fentanyl, a 1:1 dose conversion ratio was used, based on data from previous studies [15, 16]. After applying the patch, the sc administration was continued in the same dose for 6 h, after which 50 % of the dose was given during an extra 6 h [17]. After 12 h of patch application, the sc administration was stopped. Patients treated with a patch were prescribed medication for the treatment of breakthrough pain, mostly oral morphine or oxycodone in an immediate release formulation but not rapid onset opioids. For all patients, co-medication was screened for the concurrent use of strong CYP3A4 inhibitors or inducers. Also, liver function was checked based on the laboratory values of bilirubin, alanine aminotranferase (ALT), aspartate aminotransferase (AST) and albumin. The study was approved by the medical ethics review board (MEC 09.332) and conducted in accordance with the Declaration of Helsinki. Written informed consent was obtained from all participants. The trial was registered in the Dutch Trial Register (Trial registration ID: NTR4369, http://www.trialregister.nl/trialreg/admin/rctsearch.asp?Term=4369).

### Pharmacokinetic sample collection

Patients were included in the study as soon as possible after admission to the ward or after the start of fentanyl. Blood samples for pharmacokinetic analysis were taken during a maximum of 72 h after the start of fentanyl and after each change in the opioid regimen (dose, route of administration). The protocol prescribed sampling twice a day, around 8 am and 8 pm, a baseline plasma sample before every change in the regimen and a series of samples maximally once a day around the administration of an extra subcutaneous bolus at baseline, 5, 15, 30 and 60 min after administration. Samples were collected using potassium EDTA tubes. After centrifugation of the tube, the supernatant was collected and stored at −70 °C until analysis at the laboratory of Translational Pharmacology (Erasmus MC Cancer Institute).

### Measurements of fentanyl plasma concentrations

Fentanyl in plasma was quantitated using a validated UPLC-MS/MS method consisting of a Waters Acquity UPLC sample manager coupled to a triple quadruple mass spectrometer operating in the multiple reaction monitoring mode (MRM) with positive ion electrospray ionization (Waters, Etten-Leur, The Netherlands). The multiple reaction monitoring transitions was set at 337 → 188 for fentanyl and 342 → 188 for the internal standard fentanyl-d5.

Chromatographic separations were achieved on an Acquity UPLC® BEH C18 1.7 μm 2.1 × 100 mm column thermostated at *T* = 50 °C. A gradient at a flow rate of 0.350 mL/min was achieved with mobile phase A, composed of 2 mM ammonium formate and 0.1 % formic acid, and mobile phase B, composed of methanol with 0.1 % formic acid. A linear gradient was used, with 90 % mobile phase A from 0–0.50 min followed by 90–0 % mobile phase A, from 0.50 to 2 min, holding on 0 % mobile phase A (i.e. 100 % mobile phase B) for 2 min. This was succeeded by a linear gradient back to 90 % mobile phase A from 4.0 to 4.1 min, which was held for 1.9 min to re-equilibrate. The overall cycle time of the method was 6 min. The calibration curves were linear over the range of 0.100 to 10.0 ng/mL with the lower limit of quantitation validated at 0.100 ng/mL for fentanyl. The extraction of 200 μL of plasma involved a deproteinization step with 100 μL of internal standard solution in acetonitrile and 100 μL of acetone followed by a simple liquid–liquid extraction with 1-mL ethyl acetate after the addition of 100 μL of 4 % ammonium hydroxide. For fentanyl (linear calibration range 0.100–10.0 ng/mL), the within- and between-run precisions at five tested concentrations, including the lower limit of quantitation (LLQ), were ≤5.52 and ≤6.12 %, respectively, while the average accuracy ranged from 88.5 to 94.0 %. No adsorption of fentanyl was observed to the sampling and/or storing tubes. The inter-day coefficient of variation (CV) at five tested concentrations, including the LLQ, was ≤7.5 % in individual validation runs.

### Population pharmacokinetic model for fentanyl

The analysis of log-transformed concentration–time data was carried out with non-linear mixed-effects modelling in NONMEM (version 7.3; Icon Development Solutions, Hanover, MD) by means of the first-order conditional estimation method with or without eta-epsilon interaction [18]. Model building was assisted by Perl-speaks-NONMEM (PsN version 4.2.0, http://psn.sourceforge.net/) [19, 20] and the graphical evaluation with R (version 3.0.3, http://www.r-project.org/) and Xpose (version 4.4.1, http://xpose.sourceforge.net/) [21].

As a starting point, a one-compartment model with first-order absorption preceded by a lag time was used. Several model components were evaluated, including one- versus two-compartment disposition models, alternative absorption models following transdermal administration (first- versus zero-order), differences between the two administration routes in absorption parameters, i.e. absorption rate constant (*k*_a_) and lag time (*t*_lag_), and inclusion of allometrically scaled body weight on disposition parameters. Concentrations below the lower limit of quantification comprised less than 1 % of the data and were discarded from the analysis.

Inter-individual variability (IIV) in pharmacokinetic parameters was modelled using log-normal models. An occasion was defined as a transdermal dose followed by at least one observation, and inter-occasion variability (IOV) was evaluated on absorption parameters as proposed by Karlsson and Sheiner [22]:

$$ {P}_{ik} = P\ \cdotp\ {e}^{\eta i + {K}_{{}^{ik}}} $$

where *P*_*ik*_ represents the parameter *P* for the *i*th individual on occasion *k*, *P* is the typical parameter for the studied population, *η*_*i*_ is the patient-specific random effect describing the discrepancy between the typical and individual parameter and *κ*_*ik*_ is the random effect accounting for the IOV. *η*_*i*_ and *κ*_*ik*_ are assumed to be normally distributed with mean zero and estimated variance *ω*^2^ and *π*^2^, respectively.

Alternative residual error models were evaluated, including homoscedastic or heteroscedastic residual errors as well as a model combining both types of error.

### Model evaluation

The selection between alternative models during the modelling process was based on scientific plausibility and statistical significance. Statistical evaluation comprised the analysis of goodness-of-fit plots, precision of parameter estimates, condition number and the likelihood ratio test based on the change of the objective function value (OFV). The OFV is given by minus twice the log likelihood, and a difference in OFV (ΔOFV) between nested models is approximately *χ*^2^ distributed. A ΔOFV of 3.84, 6.64 and 10.8 corresponds to *p* values of 0.05, 0.01 and 0.001, respectively, when one parameter is added to the model (1 *df*). The Akaike information criterion (AIC) was used to compare non-hierarchical models. The magnitude of η- and ε- shrinkage was computed according to Karlsson and Savic [23] to judge the reliability of various diagnostic plots. The uncertainty of parameter estimates was assessed using the non-parametric bootstrap procedure in PsN (1000 bootstrap datasets). The predictive performance of the final model was evaluated with a population prediction-corrected visual predictive check (pcVPC) through 1000 simulations of the dataset [24].

## Results

### Patients

Plasma samples for pharmacokinetic analysis were available for 52 patients (Table 1). Three patients participated in the study twice. Treatment with td and sc fentanyl in relation to the observations for all patients is shown in supplemental figure 1. In 13 patients, samples were available during sc treatment without previous td administration; in 9 patients, samples were available during treatment with td fentanyl without previous or concurrent sc treatment; and in 32 patients, samples were available during treatment with sc or td fentanyl, but the other treatment route was given until shortly before sampling (semi-simultaneous treatment) or simultaneously. The majority of patients (*n* = 33) already used transdermal fentanyl before admission. In total, 942 fentanyl plasma samples were available with a median of 15 sparse samples per patient (range 1–86) and a median concentration of 1.33 ng/mL (range 0.122–10.7 ng/mL). One patient used a strong CYP3A4 inducer—carbamazepine 200 mg—during his study period. In none of the patients, the combination of AST and/or ALT above upper limit of normal (ULN), bilirubin above ULN and albumin below lower limit of normal was found, and therefore it was concluded that none of the patients had liver failure. Doses for the transdermal route varied from 12 to 400 μg/h (median 50 μg/h), and doses for the continuous subcutaneous infusion ranged from 10 to 300 μg/h (median 75 μg/h).

**Table 1:** Patient characteristics

Characteristics (*n* = 52)	No. (%)
Median age (years)—range	63 (23–80)
Sex	
Male	33 (63)
Female	19 (37)
Race	
Caucasian	47 (90)
Other	1 (2)
Unknown	4 (8)
WHO performance status	
0	0
1	19 (37)
2	17 (33)
3	4 (8)
Unknown	12 (23)
Median body mass index—range	25 (18–40)
Median NRS in rest at start of fentanyl or on admission—range	5 (2–10)
Primary tumour localization	
Breast	8 (15)
Colorectal	5 (10)
Prostate	7 (13)
Soft tissue sarcoma/GIST	6 (12)
Urinary tract (including the kidney)	8 (15)
Other	18 (35)
Median albumin—range	39 (29–49)
Median AST (U/l)—range	31 (13–216)
Median ALT (U/l)—range	22 (7–131)
Median total bilirubin (μmol/L)—range	7 (3–16)

### Fentanyl pharmacokinetics

The pharmacokinetics of fentanyl-administered sc and td were best described by a one-compartment model with first-order elimination and separate first-order absorption processes for each route. The residual error was most adequately described by a heteroscedastic model parameterised as an additive model on the log-scale. Due to the sparse sampling design, we were unable to estimate all model parameters satisfactorily, particularly with respect to parameters describing the absorption part. Hence, the apparent volume of distribution (*V*/*F*) was fixed to 280 L [25]. A sensitivity analysis carried out with values of *V*/*F* ±50 % fixed in 10 % increments showed the model to be insensitive to the value and other parameter estimates to be stable within the tested range, with only *t*_lag_ and *k*_a,sc_ varying slightly (less than ±25 % deviation from the final PK parameter values). Inclusion of allometrically scaled body weight on CL/*F* and *V*/*F* was found to explain some variability and was kept to increase model stability. The final population model parameters including bootstrap results are presented in Table 2.

**Table 2:** Typical population pharmacokinetic parameter estimates for subcutaneous and transdermal fentanyl and bootstrap analysis results

Parameter (units)	NONMEM estimate (%RSE)^a^		Bootstrap mean (95 % CI)^b^	
Structural model parameters				
*k*_a_ subcutaneous (h^−1^)	0.0358	(24.4)	0.0374	(0.0248, 0.0555)
*t*_lag_ transdermal (h)	4.73	(21.2)	4.65	(2.25, 6.98)
*k*_a_ transdermal (h^−1^)	0.0135	(16.8)	0.0140	(0.0105, 0.0188)
*V*_70kg_/*F* (L)^c^	280	(fix)	–	
CL_70kg_/*F* (L h^−1^)^d^	49.6	(9.36)	50.4	(40.9, 61.6)
Inter-individual variability (%CV)				
*k*_a_ subcutaneous	93.5	(15.2^e^)	91.1	(59.6, 119)
*F* transdermal	42.3	(30.0^e^)	45.7	(19.7, 67.8)
*k*_a_ transdermal	42.4	(23.9^e^)	41.4	(10.5, 59.2)
CL/*F*	43.2	(15.2^e^)	41.6	(27.1, 53.9)
Inter-occasion variability (%CV)				
*k*_a_ transdermal	32.8	(51.1^e^)	39.2	(12.0, 77.0)
Residual unexplained variability (%CV)				
Proportional residual error	23.4	(5.17^f^)	23.2	(20.6, 25.6)

^a^The condition number of the final model was 24.99

^b^Mean and 95 % bootstrap percentile confidence intervals. Runs with estimates near a boundary (*n* = 150), rounding errors (*n* = 165) or crashed (*n* = 3) were skipped when calculating results

^c^ *V*_70kg_/*F* = 280 × (WT/70)

^d^CL_70kg_/*F* = estimate × (WT/70)^0.75^

^e^%RSE is reported on the approximate standard deviation scale (standard error/variance estimate)/2. η-shrinkage for inter-subject variability ranged between 14.6 and 48.4 % and η-shrinkage for inter-occasion variability was >35 %

^f^ε-shrinkage was 5.97 %

*CI* confidence interval; *CL*_*70kg*_/*F* apparent clearance for a subject with 70 kg; %*CV* percent coefficient of variation, reported as sqrt(variance) × 100 %; *F* bioavailability; *k*_*a*_ absorption rate constant; %*RSE* relative standard error; *t*_*lag*_ absorption lag time; *V*_*70kg*_/*F* apparent volume of distribution for a subject with 70 kg; *WT* weight (kg)

The estimated population value for CL/*F* in a 70-kg subject was 49.6 L/h. The estimation of a *t*_lag_ for td administration led to an improvement of the model fit (*p* value <0.001) with the final value of 4.73 h. In contrast, the inclusion of a *t*_lag_ was not relevant for sc administration. The model was compared with a model with zero-order absorption for td fentanyl, and the AIC was clearly in favour of the first-order absorption (AIC more than 60 points lower). The estimated absorption rate constant for subcutaneous fentanyl was 0.0358 h^−1^ and for transdermal fentanyl 0.0135 h^−1^.

**Fig. 1:**
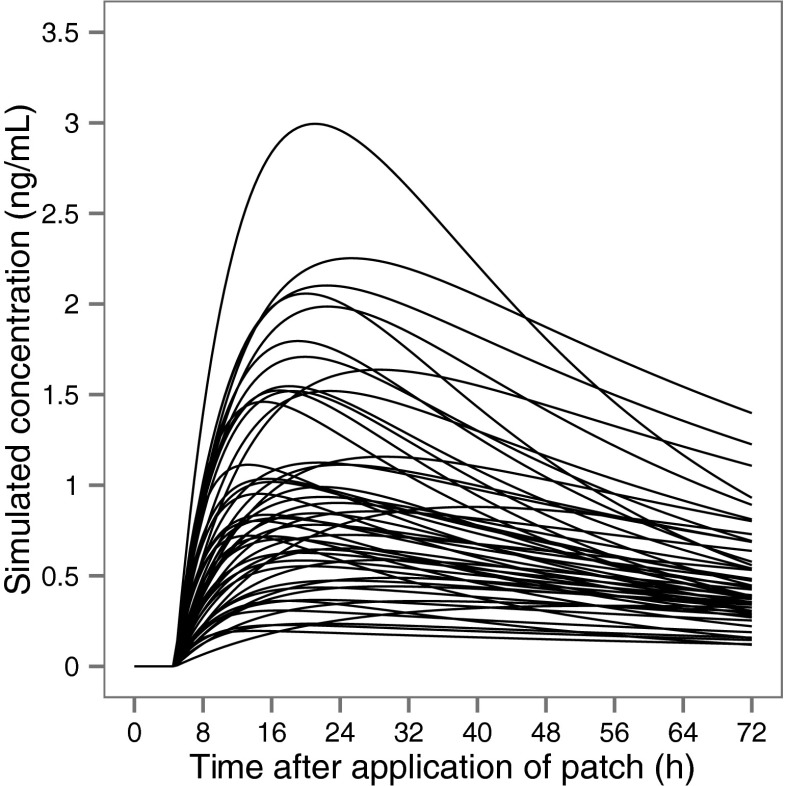
Stochastic simulation of fentanyl plasma concentrations versus time after application of a transdermal patch with a delivery rate of 50 μg/h in 52 patients

IIV was included on *k*_a_ for both routes (93.5 and 42.4 % for sc and td, respectively), td bioavailability and apparent clearance (CL/*F*). Bioavailability of td fentanyl was allowed to differ between individuals with an estimated variability of 42.3 %. IOV on td *k*_a_ resulted in a significant improvement of the model (*p* < 0.01) with an estimated value of 32.8 %. The consequence for rate and extent of absorption following td administration, given these characteristics, is illustrated in Fig. 1.

**Fig. 2:**
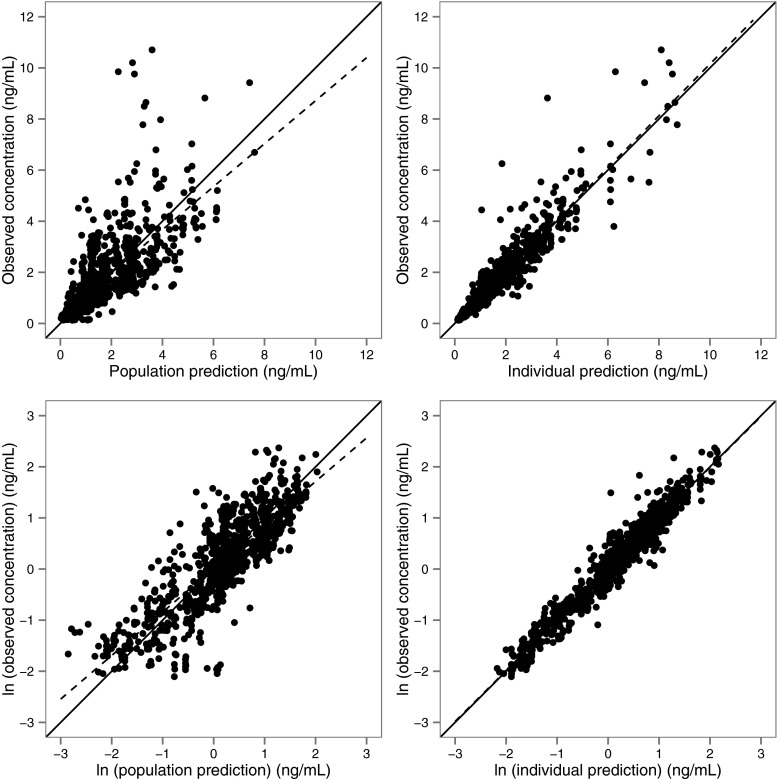
Goodness-of-fit plots for the final model. Observed fentanyl plasma concentrations versus population predictions (*left panels*) and individual predictions (*right panels*) in normal (*top panels*) and logarithmic scale (*bottom panels*). The *solid line* represents the line of identity (*x* = *y*) and the *dashed line* represents a linear regression line

**Fig. 3:**
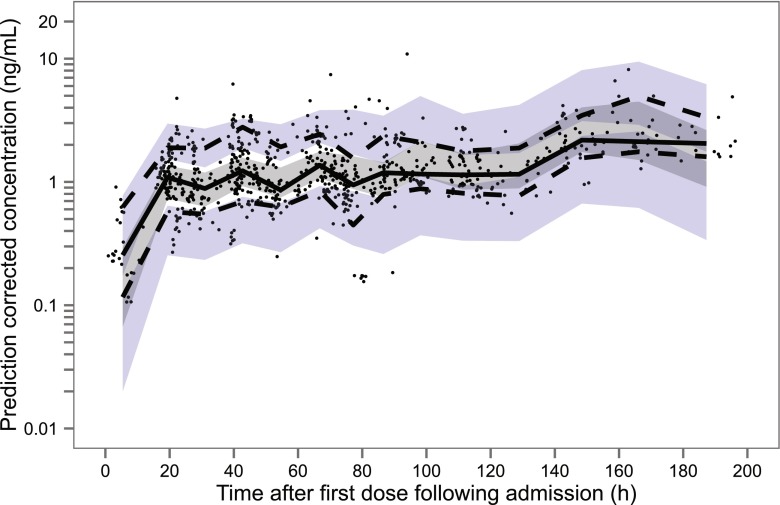
Population prediction-corrected visual predictive check for the final model for subcutaneous and transdermal fentanyl. The *x*-axis represents the time after the first recorded dose of fentanyl after admission. *Dots* are the population predicted-corrected individual observations, and the *solid* and *dashed lines* represent the median and the 10th and 90th percentiles of the observed data, respectively. The *shaded areas* represent the simulation-based 95 % confidence interval for the simulated data percentiles

The model was found to describe the observed concentrations well (Fig. 2). The performance of the model to predict median concentrations was good as illustrated by a pcVPC shown in Fig. 3. Additional goodness-of-fit plots can be found in Supplemental data.

### Evaluation of rotations from subcutaneous to transdermal fentanyl

For 14 patients, multiple plasma samples were available shortly before and after rotation from sc to td fentanyl using the 12 h scheme. In 12 of these patients, a rise in plasma fentanyl concentrations was seen after application of the first patch. Furthermore, the intensity of side effects increased in 9 patients while in 3 patients, severe fentanyl-related toxicity occurred, necessitating adjustment of treatment. The severe toxicity consisted of respiratory depression, severe drowsiness and nausea.

**Fig. 4:**
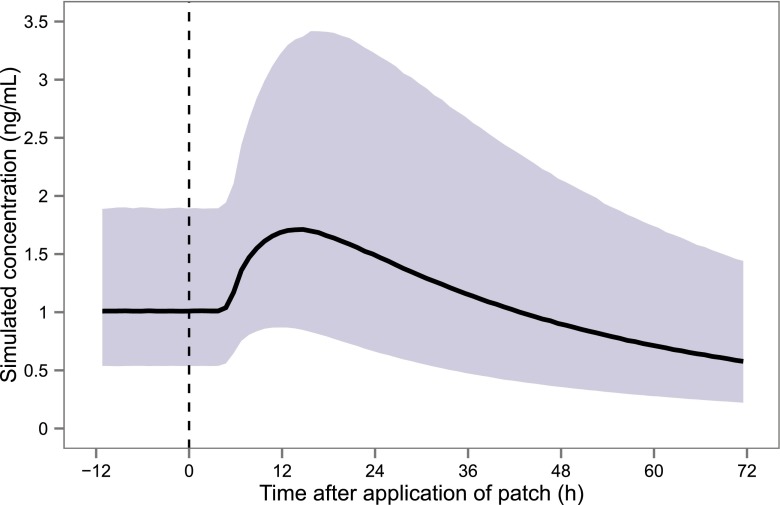
Simulated fentanyl plasma concentrations during the rotation from a subcutaneous infusion of 50 μg/h at steady state to a transdermal patch with a delivery rate of 50 μg/h using the 12-h scheme (1000 simulations of 52 subjects). Following this scheme, the subcutaneous administration is continued in the same dose for 6 h after applying the transdermal patch, after which 50 % of the dose is given during an extra 6 h. The simulated *solid line* represents the median of the simulated data, and the *shaded area* represents the 80 % prediction interval. The *vertical dashed line* represents the time of patch application

By using the final model, fentanyl plasma concentrations expected around and after rotation were predicted for a population of 52 patients through stochastic simulation. Figure 4 illustrates plasma fentanyl concentrations during the rotation from a sc infusion of 50 μg/h to a td patch with a delivery rate of 50 μg/h using the 12-h scheme. After the application of the td patch, the simulated median peak concentration is higher than the steady-state concentration of subcutaneous fentanyl. In addition, concentrations immediately after the end of the rotation scheme, i.e. 12 h after the application of the patch, are very variable with the 10th and 90th percentiles equal to 0.87 and 3.22 ng/mL (median value 1.68 ng/mL). Simulated fentanyl plasma concentrations using a 6-h scheme [26] produced similar results, and comparative plots can be found in Supplemental data.

## Discussion

This prospective study in Caucasian cancer patients treated with fentanyl provides us with new insights into the pharmacokinetics of fentanyl which are relevant for clinical practice.

Firstly, we developed a population pharmacokinetic model for sc and td fentanyl from a high number of sparse samples in this patient cohort. We found that a one-compartment model adequately describes the pharmacokinetics of sc and td fentanyl, similarly to the results of previous studies with td fentanyl [27, 28]. We were able to distinguish inter-individual variability between absorption and elimination pharmacokinetic parameters that along with inter-occasion and residual variability explain the high variability in plasma concentrations and possibly also clinical effects.

Similar PK models have been described following td administration previously [27, 28]. In our study, the CL/F was estimated to 49.6 L/h, which is similar to the values of 40.8 and 42.4 L/h obtained in previous PK studies [10, 29]. Furthermore, in line with previous models, the absorption from td patches over 72 h was found to be closer to a first-order than to a zero-order process, with a potential to lead to fluctuations in plasma concentrations during treatment. Indeed, fluctuation in plasma concentrations has been reported in several studies [30–33]; however, the clinical relevance of this finding was never widely acknowledged. In clinical practice, however, many patients report either lower pain scores and/or more side effects after patch change, and on the other hand, for worsening of pain during the third day, a patch is used [16, 34].

The estimated absorption rate constant and absorption lag time are in agreement with the values found by Bista et al. [28] (0.013 h^−1^, *k*_a_) and Kokubun et al. [27] (0.0145 h^−1^ and 4.93, *k*_a_ and lag time) for td fentanyl. Such slow absorption relative to elimination (absorption and elimination half-lives 51.3 and 3.91 h, respectively) results in that the decline in plasma concentrations after achieving the peak following transdermal administration reflects absorption rather than elimination. The *T*_max_ predicted by our model in a typical patient was about 20.5 h after the administration of a patch. This value is known to vary substantially between patients and values in the range 12–48 h have been reported [35]. The td absorption with large variability is illustrated in Fig. 1.

For sc fentanyl, published PK data are limited. In the only other study in patients treated with continuous infusion of sc fentanyl, only one plasma sample was taken showing considerable variability, but no PK parameters were presented [36]. Capper et al. [37] described the pharmacokinetics of fentanyl after a bolus of 200 μg fentanyl sc in nine healthy volunteers and reported a CL/*F* of 53.7 L/h, similar to our estimate, and a rapid absorption (*T*_max_ 10–30 min). We found a slow absorption with substantial IIV in a situation in which fentanyl dosages were titrated using continuous infusion with extra boluses as needed for pain control. The estimation of a separate *k*_a_ following sc boluses was tested but not supported by the data. In addition, the model was evaluated with a fast absorption process following sc administration by fixing *k*_a_ for this route (2 h^−1^). However, goodness-of-fit plots and the fit of the model was statistically significantly worse (*p* < 0.001). In four patients in our study, plasma samples were available after stopping sc fentanyl because of rotation to another type of opioid. In all, a slow decrease in fentanyl plasma concentrations was noticeable which supports our data. It may be that also after subcutaneous treatment, some subcutaneous dose depot is formed, as has been reported for td fentanyl [9], but there are no firm data following sc infusion. Thus, our model describes sc infusion data, but mechanistic conclusions should not be drawn. However, if the slow absorption would be that slow, it suggests that continuous fentanyl is less suitable for fast titration.

High to moderate variability in PK parameters and plasma concentrations has been reported before for td fentanyl, but literature on sc fentanyl is scarce. Kokubun et al. and Bista et al. [28] estimated moderate IIV on CL/*F* to 43.5 and 38.5 %, respectively, following td patches. Although there are differences in patch type (reservoir versus matrix) and study populations, i.e. regarding the amount of sc fat/body mass index and hepatic metabolism, IIV was in agreement with our estimate of 43.2 %. The IIV on *k*_a_ in the study of Kokubun was substantially greater (71.9 %) than the 42.4 % we obtained, but we also found different occasions as a significant source of variability (IOV, 32.8 %). Other studies have reported substantial variation in bioavailability (range 60 to 97 %), in the measured rate of absorption (e.g. 12.5 to 60.4 μg/h with a patch of 50 μg/h) [29] and in inter- and intra-subject variability in plasma fentanyl concentrations (50.7 and 34.4 %, respectively) [30].

Lastly, this is to our knowledge the first evaluation of rotations from sc to td fentanyl, using the scheme described by Kornick et al. [17] who studied rotations from the intravenous (iv) to the transdermal route. More recently, a scheme using a two-step taper of iv fentanyl in 6 h was found to be safer than the 12-h method [26]. In a PK study by the same group, using the 6-h scheme, a rise in plasma concentrations was seen after 3 h but without adverse effects [38]. According to the current study, the use of the 12-h scheme and a 1:1 dose conversion may lead to a rather steep rise in plasma concentrations for some patients and clinically evident toxicity. Based on the final model, we simulated rotations using the 6-h scheme. This scheme may also lead to a rise in plasma levels and therefore potential toxicity. This is probably caused by the fact that plasma concentrations fall slower after stopping a sc administration than after an iv administration and by the finding that absorption following td administration appears to follow a first-order process. For confirmation of our findings, we have planned a prospective pharmacokinetic evaluation study of different rotation schemes without overlap of routes and with or without dose reduction of the first patch.

Strengths of our study are the longitudinal data that we assembled in one patient cohort and the large number of samples available for PK analysis. One limitation in our study was that, although we were able to estimate IIV and IOV variability in PK parameters, due to a limited sample size, we did not investigate possible sources of variability through covariate modelling. Furthermore, due to semi-simultaneous administration following different routes of administration, the observed concentrations were the sum of those obtained following each route. Especially, many patients started on sc fentanyl after hospital admission while they already used fentanyl td at home, and sc bolus injections for rescue were frequently administered over the full study period. Although the semi-simultaneous administration was accounted for in modelling, the study design was not optimal for modelling purposes.

In conclusion, this study describes the pharmacokinetics of sc and td fentanyl in one patient cohort. Findings relevant for clinical practice are the moderate to large IIV and IOV and that absorption following td administration potentially may lead to fluctuations in plasma concentrations. Furthermore, published rotation schemes for rotations from intravenous to transdermal fentanyl might not be applicable on rotations from subcutaneous to transdermal fentanyl.

## Electronic supplementary material

Fig. 1 Treatment with transdermal and subcutaneous fentanyl in relation to the observations for all patients. (JPG 218 kb)

High resolution image (EPS 1714 kb)

Fig. 2 Additional goodness-of-fit plots for the final model. Conditional weighted residuals versus time after the first recorded dose of fentanyl following admission (upper left panel), conditional weighted residuals versus population predictions (upper right panel) and absolute individual weighted residuals versus individual predictions (lower left panel). The grey line is a tendency line. (JPG 79 kb)

High resolution image (EPS 85 kb)

Fig. 3 Simulated fentanyl plasma concentrations during the rotation from a subcutaneous (sc) infusion of 50 μg/h at steady-state to a transdermal (td) patch with a delivery rate of 50 μg/h using the 12-h scheme (left panel) and the 6-h scheme (right panel). In the 12-h scheme, the sc administration is continued in the same dose for 6 h after applying the td patch, after which 50 % of the dose is given during an extra 6 h. In the 6-h scheme, the sc administration is continued in the same dose for 3 h after applying the td patch, after which 50 % of the dose is given during an extra 3 h. The vertical dashed lines represent the start and the end of the rotation scheme. The simulated solid line represents the median of the simulated data and the shaded area represents the 80 % prediction interval (1,000 simulations of 52 subjects). (PDF 9 kb)
