# Supplementary figures and images for: Treatment with subcutaneous and transdermal fentanyl: results from a population pharmacokinetic study in cancer patients
Source: Eur J Clin Pharmacol. 2016 Jan 14;72:459–67. doi: 10.1007/s00228-015-2005-x (PMC4792338; doi:10.1007/s00228-015-2005-x)

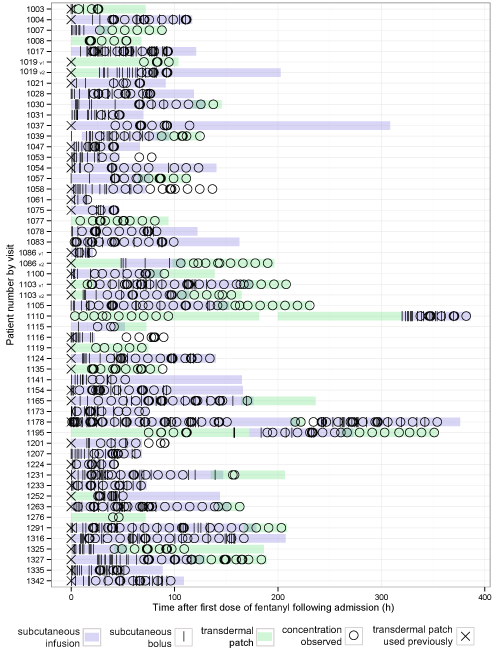

Supplement: Supplementary file 1 — Treatment with transdermal and subcutaneous fentanyl in relation to the observations for all patients. (JPG 218 kb) [file 228_2015_2005_Fig5_ESM.jpg]

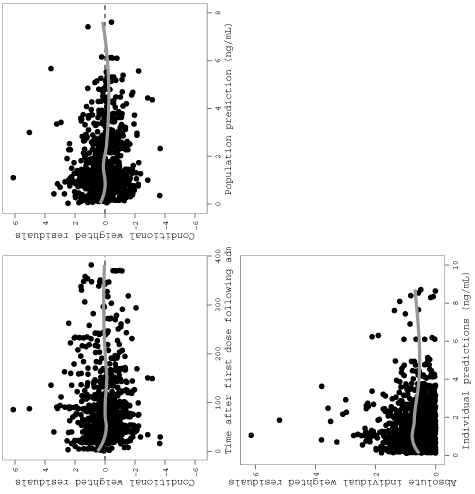

Supplement: Supplementary file 3 — Additional goodness-of-fit plots for the final model. Conditional weighted residuals versus time after the first recorded dose of fentanyl following admission (upper left panel), conditional weighted residuals versus population predictions (upper right panel) and absolute individual weighted residuals versus individual predictions (lower left panel). The grey line is a tendency line. (JPG 79 kb) [file 228_2015_2005_Fig6_ESM.jpg]

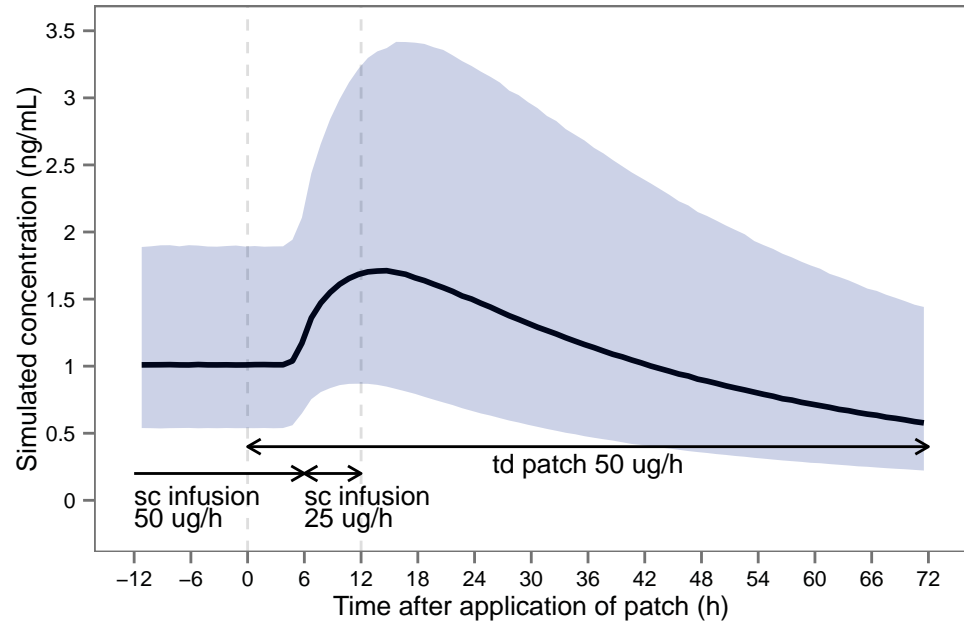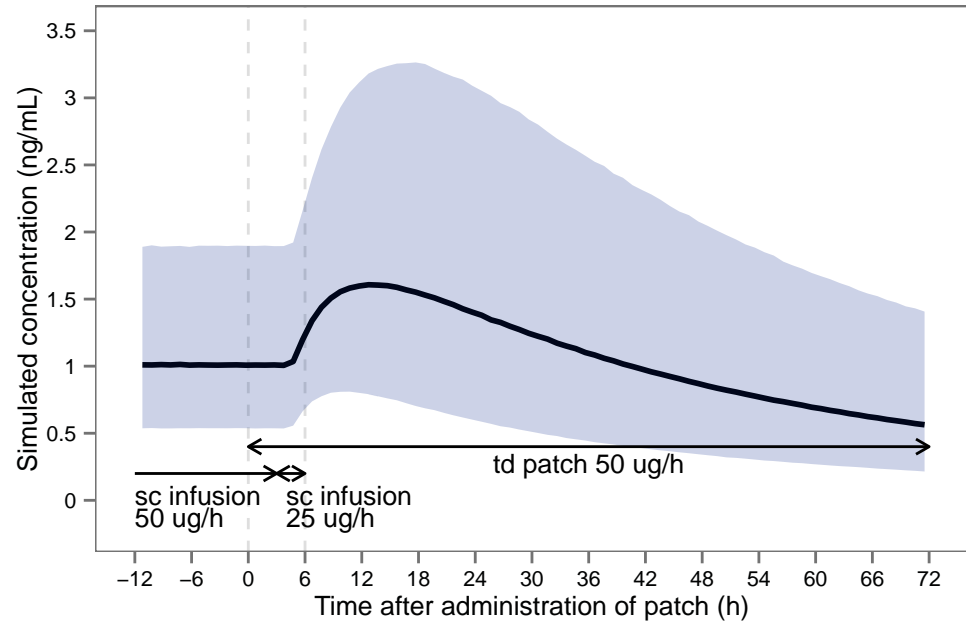

Supplement: Supplementary file 5 — Simulated fentanyl plasma concentrations during the rotation from a subcutaneous (sc) infusion of 50 μg/h at steady-state to a transdermal (td) patch with a delivery rate of 50 μg/h using the 12-h scheme (left panel) and the 6-h scheme (right panel). In the 12-h scheme, the sc administration is continued in the same dose for 6 h after applying the td patch, after which 50 % of the dose is given during an extra 6 h. In the 6-h scheme, the sc administration is continued in the same dose for 3 h after applying the td patch, after which 50 % of the dose is given during an extra 3 h. The vertical dashed lines represent the start and the end of the rotation scheme. The simulated solid line represents the median of the simulated data and the shaded area represents the 80 % prediction interval (1,000 simulations of 52 subjects). (PDF 9 kb) [file 228_2015_2005_MOESM3_ESM.pdf]
